# A Low-Cost CMOS-MEMS Piezoresistive Accelerometer with Large Proof Mass

**DOI:** 10.3390/s110807892

**Published:** 2011-08-11

**Authors:** Mohd Haris Md Khir, Peng Qu, Hongwei Qu

**Affiliations:** Department of Electrical and Computer Engineering, Oakland University, Rochester, MI 48309, USA; E-Mail: pqu@oakland.edu

**Keywords:** CMOS-MEMS, piezoresistive, polysilicon, deep reactive ion etching (DRIE)

## Abstract

This paper reports a low-cost, high-sensitivity CMOS-MEMS piezoresistive accelerometer with large proof mass. In the device fabricated using ON Semiconductor 0.5 μm CMOS technology, an inherent CMOS polysilicon thin film is utilized as the piezoresistive sensing material. A full Wheatstone bridge was constructed through easy wiring allowed by the three metal layers in the 0.5 μm CMOS technology. The device fabrication process consisted of a standard CMOS process for sensor configuration, and a deep reactive ion etching (DRIE) based post-CMOS microfabrication for MEMS structure release. A bulk single-crystal silicon (SCS) substrate is included in the proof mass to increase sensor sensitivity. In device design and analysis, the self heating of the polysilicon piezoresistors and its effect to the sensor performance is also discussed. With a low operating power of 1.5 mW, the accelerometer demonstrates a sensitivity of 0.077 mV/g prior to any amplification. Dynamic tests have been conducted with a high-end commercial calibrating accelerometer as reference.

## 1. Introduction

The piezoresistive effect is one of the most exploited physical effects in sensors. Since the discovery of the excellent mechanical properties and late on the manufacturability of silicon and other semiconductors, a large array of MEMS piezoresistive sensors have been developed [1–3]. MEMS piezoresistive accelerometers have also been extensively attempted [4]. According to the topological configurations of the sensing elements, the reported piezoresistive accelerometers can be classified into the following four thing listed categories [5]:

1. Single-clamped beams, where the seismic mass is suspended with only one beam, and the bending of the beam leads to stress and strain on both sides of the beam with opposite polarity; whereas the neutral axis itself is stress free;
2. Double-clamped beams, where the seismic mass is suspended by two (or more) beams and the mechanical stress is mainly concentrated in four areas in each beam;
3. Axially loaded beams, where the seismic mass is accelerated in the axial direction of the beam, and the acceleration leads to a stretching of the beam instead of a bending;
4. Some special sensing structures with combination of the above configurations, including asymmetrical beams [6,7].

CMOS-MEMS technology provides a viable means for monolithic integration of MEMS elements with mainstream CMOS electronics, for an overall improved device performance and possible lower cost [8]. Post-CMOS MEMS has been proven as a strong competitor in the integration [9]. Numerous CMOS-MEMS piezoresistive accelerometers using these above four topologies have been investigated. Thus far most of the reported CMOS-MEMS piezoresistive accelerometers use thin film structures as proof mass [10,11]. Although the surface micromachining processes employed for the creation of these devices are quite simple, due to the small structure thickness, the devices suffer from low sensitivity and other shortcomings. Moreover, the residual stress in the CMOS thin films often causes large structure curling. Thus, the area and mass of the proof mass structure is also limited. DRIE based dry bulk CMOS-MEMS technology has paved pathways for microfabrication of various devices with robust MEMS structures and desired device sizes [12,13]. Compared with the wet process for SCS proof mass manufacturing [14,15], the DRIE method is more effective and environmentally-friendly.

In this work, a low cost, high sensitivity CMOS-MEMS piezoresistive accelerometer is designed, fabricated and tested. Compared to the reported devices, higher sensor sensitivity and larger process tolerance are achieved by using a maskless bulk DRIE CMOS-MEMS microfabrication to include SCS as proof mass. Inherent CMOS SiO_2_/Aluminum laminated composite layers are employed to form cantilevers in which the CMOS polysilicon layer as the sensing elements are embedded. On the other end of the cantilevers, a large proof mass with SCS attached is connected to increase the stress induced in the cantilevers upon the application of an out-of-plane acceleration. Using multiple CMOS metal layers, the four poly resistors are conveniently wired to form a full sensing Wheatstone bridge for even higher sensitivity.

## 2. Device Design and Simulation

### 2.1. Device Design

**Figure 1.**
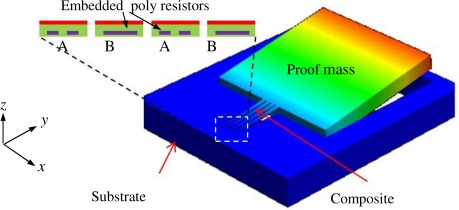
3D model of the piezoresistive sensor showing the embedded polysilicon resistors in the composite beams.

**Figure 2.**
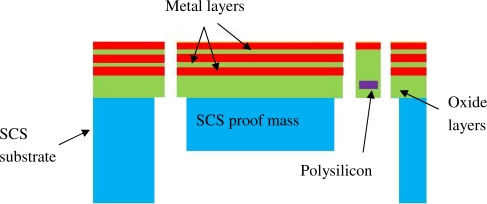
Schematic cross-section of a released sensor showing the CMOS thin films and their relative locations.

Figure 1 shows a 3-D structural model of the fabricated accelerometer with the inset showing the cross-sectional view of the CMOS composite beams in which the sensing polysilicon piezoresistors are embedded. Inset “A” and “B” show cross-sections of the folded polysilicon resistors that are arranged in longitudinal and transverse direction, respectively. The sensor has a SCS proof mass of 500 μm × 500 μm in size and approximately a 40 μm thickness. The SCS proof mass is anchored through the four cantilever beams that consist of the inherent CMOS SiO_2_/Al thin films with a total thickness of approximately 5 μm. The planar dimension of each composite beam is 200 μm × 13 μm. The inherent CMOS polysilicon layer, which is used as the piezoresistive sensing material, has a thickness of approximately 0.35 μm, according to the standard ON 0.5 μm CMOS technology used in this project. The schematic cross-section of the CMOS thin films and their spatial locations are illustrated in Figure 2. The typical thin film parameters of the ON 0.5 μm technology is listed in Table 1 [16].

When the sensor is subject to an out-of-plane motion, the induced stress on the longitudinal and transverse polysilicon resistors will result in the relative change of resistance *δR/R* given by:

(1) $$\frac{\Delta R}{R}={\left(\frac{\Delta R}{R}\right)}_{l}=G_{\mathrm{polyl}}{ɛ}_{x}$$

where the subscripts “*l*” and “*t*” denote the longitudinal and transverse relative change of resistance, respectively; *v* is the Poisson’s ratio of the silicon having the value of 0.27; *G_polyl_* and *G_polyt_* are the longitudinal and transverse gauge factors. In the ON 0.5 μm technology used in this work, the polysilicon layer has a nominal sheet resistance *ρ_s_* of 26.1 Ω/□ [16], which is equivalent to a resistivity of 9.14 × 10^−4^ .cm and a boron doping concentration of 1.42 × 10^19^ cm^−3^. This amount of doping concentration corresponds to estimated longitudinal and transverse gauge factor of 40 and −15, respectively [3]. The resistivity of the polysilicon can be determined using:

(2) $$\rho_{\mathrm{poly}}=\rho_{s}t_{\mathrm{poly}}$$

(3) $$\frac{\Delta R}{R}={\left(\frac{\Delta R}{R}\right)}_{t}=G_{\mathrm{polyt}}{ɛ}_{y}$$

where *t_poly_* is 0.35 μm. The relationship between the axial (y-direction) and transverse strains can be evaluated using the following equation:

(4) $${ɛ}_{t}=-\nu {ɛ}_{x}$$

The axial strain, *ε_x_* in the direction of *x*, which occurs on the composite beam is given by:

(5) $${ɛ}_{y}=\frac{z}{R_{c}}$$

where *z* is the distance from the neutral axis; and *R_c_* is the radius of curvature of the bending beam. The Young’s modulus of aluminum and silicon dioxide (SiO_2_) material are close. According to the ON 0.5 μm technology, the total thickness of the cantilever is approximately 5.3 μm, with the 0.35 μm polysilicon being located above the 0.4 μm field oxide at the bottom of the cantilever [16]. As the 0.4 μm thickness is much smaller than the half thickness of the beam, it is acceptable to assume that the neutral axis is at the center of the cantilever beam. The beam bending moment can be obtained by the integration of the stress through the thickness of the beam, *H_b_* and is given by [17]:

(6) $$M=\int_{-H_{b}/2}^{H_{b}/2}\left[(W_{b}\mathrm{dz})\sigma_{x}\right]\cdot z$$

where *σ_x_* is the axial stress of the beam. Since the relation between the stress and strain is *σ_x_* = *E*_ε*_x_*_:

(7) $$M=\int_{-H_{b}/2}^{H_{b}/2}\left[(W_{b}dz)\frac{Ez^{2}}{R_{c}}\right]=\frac{EW_{b}}{R_{c}}\int_{-H_{b}/2}^{H_{b}/2}z^{2}dz=\frac{EW_{b}H_{b}^{3}}{12R_{c}}$$

which can be further simplified to:

(8) $$\frac{1}{R_{c}}=\frac{12M}{EW_{b}H_{b}^{3}}=\frac{M}{\mathrm{EI}}$$

where *I* is the beam moment of inertia and is given by:

(9) $$I=\frac{W_{b}H_{b}^{3}}{12}$$

It should be noted that due to the composite nature of the cantilevers, the Young’s Modulus *E* in the above equations is the effective value of the beams that are comprised of SiO_2_ and top aluminum layer in the CMOS stack. Since the SiO_2_ is much thicker than the Al layer in the composite beam, and the Young’s Modulus of SiO_2_ is slightly greater than aluminum’s, *E* can be approximated as SiO_2_ Young’s Modulus of 70 GPa [17]. The same *E* applies to the following derivations.

**Figure. 3.**
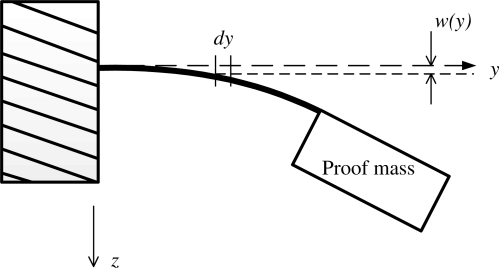
Illustrative deformation of the sensing structure subject to a downward acceleration.

Referring to Figure 3, the solution of the bending beam with proof mass results in the maximum bending stress at the beam support to substrate (*y* = 0), which is given by:

(10) $$\frac{1}{R_{c}}=\frac{d^{2}w}{dy^{2}}=\frac{M}{\mathrm{EI}}=\frac{\mathrm{ma}}{\mathrm{EI}}\left(L_{b}+\frac{L_{\mathrm{pm}}}{2}\right)$$

and from Equation (5), the strain occurs at the top (tension) and bottom (compression at polysilicon layer) of the beam surface is:

(11) $${ɛ}_{\mathrm{poly}}=\frac{z}{R_{c}}=\frac{H_{b}}{2R_{c}}$$

Inserting Equation (10) to Equation (11) yields:

(12) $${ɛ}_{\mathrm{poly}}=\frac{H_{b}}{2}\frac{\mathrm{ma}}{\mathrm{EI}}\left(L_{b}+\frac{L_{\mathrm{pm}}}{2}\right)=\frac{6m}{EW_{b}H_{b}^{2}}\left(L_{b}+\frac{L_{\mathrm{pm}}}{2}\right)a$$

The complete solution of the relative change of resistance due to the acceleration applied to the four cantilever beams can be then computed by substituting Equation (12) into Equations (1) and (2), which yields:

(13) $${\left(\frac{\Delta R}{R}\right)}_{l}=G_{\mathrm{polyl}}\frac{1.5m}{{\mathrm{EW}}_{b}H_{b}^{2}}\left(L_{b}+\frac{L_{\mathrm{pm}}}{2}\right)a$$

(14) $${\left(\frac{\Delta R}{R}\right)}_{t}=G_{\mathrm{polyt}}\frac{1.5m\nu }{{\mathrm{EW}}_{b}H_{b}^{2}}\left(L_{b}+\frac{L_{\mathrm{pm}}}{2}\right)a$$

(15) $$R=\rho_{\mathrm{poly}}\frac{L_{\mathrm{poly}}}{W_{\mathrm{poly}}t_{\mathrm{poly}}}=\rho_{s}\frac{L_{\mathrm{poly}}}{W_{\mathrm{poly}}}$$

where *R* is the original resistance of each poly resistor without acceleration, which is calculated to be 1.1 kΩ using Equation (15). The resonant frequency, *f* of the sensor is calculated as 1.85 kHz using the equation given by:

(16) $$f=\frac{1}{2\pi }\sqrt{\frac{k}{m}}$$

where *k* is the stiffness coefficient of the composite beam calculated to be 14.21 N·m^−1^ which is defined as:

(17) $$k=\frac{EW_{b}H_{b}^{3}}{4L_{b}^{3}}$$

Other parameters such as the sensor geometric and material properties used in Equations (1) and (2) and their values are given in Table 2.

**Figure 4.**
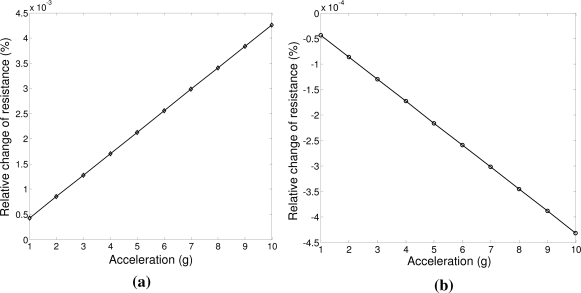
**(a)** Coventor simulation results of piezoresistance change in longitudinal direction as a function of out-of-plane acceleration; and **(b)** Piezoresistance change in transverse direction versus out-of-plane acceleration.

Using Equations (13) and (14), the longitudinal and transverse relative change of resistance with acceleration from 1 g to 10 g are estimated and the results are shown in Figure 4. The calculation results show that the longitudinal relative change of the piezoresistance +4.26 × 10^−4^ %/g or +4.3 mΩ/g, while the transverse relative change of the piezoresistance has a sensitivity of −0.46 × 10^−4^ %/g or −0.46 mΩ/g.

### 2.2. Self Heating Effect of the Piezoresistors

**Figure 5.**
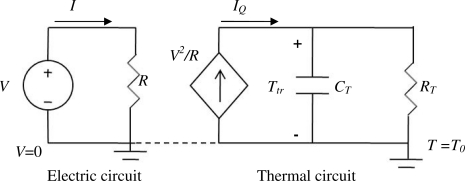
Circuit model for the self heating of a resistor driven by a voltage source.

Self heating effect of the polysilicon resistors is estimated to evaluate its impact on the performance of the sensor fabricated particularly using ON Semiconductor 0.5 μm CMOS technology used in this project. When a biasing voltage is applied to a polysilicon piezoresistor, the Joule heat generated will raise the temperature of the resistor which consequently changes the sensing resistance. The resistance change caused by this self heating is superposed to the sensing resistance caused by the acceleration to be measured. The lumped-element thermal circuit in Figure 5 can be used to estimate the temperature rise and in turn the resistance variations resulted from the Joule heating.

In Figure 5, the electric circuit consists of a voltage source and a resistor which is the original resistance of the polysilicon piezoresistors. The thermal circuit consists of three elements: the diamond shape represent a dependent current source that provides the Joule heat power *V^2^/R*; the capacitor *C_T_* represents the heat capacity of the resistor; and the resistor *R_T_* represents the heat resistance from the polysilicon resistor to the thermal ground—the substrate on which the piezoresistive cantilevers are attached. *T_0_* = *T_R_* is the substrate temperature which is assumed as the room temperature of 25 °C or 293 K. In the thermal circuit, the current (heat flux) is denoted as *I_Q_*. The relative change of resistance due to self heating effect in the polysilicon resistor can be then estimated as:

(18) $${\left(\frac{\Delta R}{R}\right)}_{\mathrm{thermal}}=\alpha_{\mathrm{poly}}(T_{\mathrm{ss}}-T_{R})=\alpha_{\mathrm{poly}}\Delta T$$

where *α_poly_* is the temperature coefficient of resistance (TCR) of polysilicon as listed in Table 2; *T_SS_* represents the final steady state temperature of the sensing element. The transient response of the temperature, *T_tr_* is derived as:

(19) $$T_{\mathrm{tr}}=T_{R}+\frac{R_{T}V^{2}}{R_{R}\left(1+\frac{\alpha_{\mathrm{poly}}R_{T}V^{2}}{R_{R}}\right)}\left(1-e^{-\left(\frac{1}{R_{T}C_{T}}\right)t}\right)$$

*R_R_* = *R* = 1.1 kΩ is the designed resistance of the polysilicon resistor at room temperature and is calculated using Equation (15). Due to the great thermal conductivity difference among the thin films in the system and the layer sequence in the cantilevers, it is assumed that the heat flux generated on the polysilicon resistors travels in the following path: polysilicon resistors → SiO_2_ layer → metal 3 layer → substrate. The corresponding thermal resistance, *R_T_* and capacitance, *C_T_* can be calculated using Equations (20) and (21) as:

(20) $$R_{T}=R_{\mathrm{Tpoly}}+R_{\mathrm{TSiO}2}+R_{\mathrm{TAl}}=\frac{H_{\mathrm{poly}}}{\kappa_{\mathrm{poly}}A_{\mathrm{poly}}}+\frac{H_{\mathrm{ox}}}{\kappa_{\mathrm{ox}}A_{\mathrm{ox}}}+\frac{L_{\mathrm{Al}}}{\kappa_{\mathrm{Al}}W_{\mathrm{Al}}H_{\mathrm{Al}}}$$

(21) $$\frac{1}{C_{T}}=\frac{1}{C_{\mathrm{Tpoly}}}+\frac{1}{C_{\mathrm{TSiO}2}}+\frac{1}{C_{\mathrm{TAl}}}$$

The values used and calculated are listed in Table 3.

**Figure 6.**
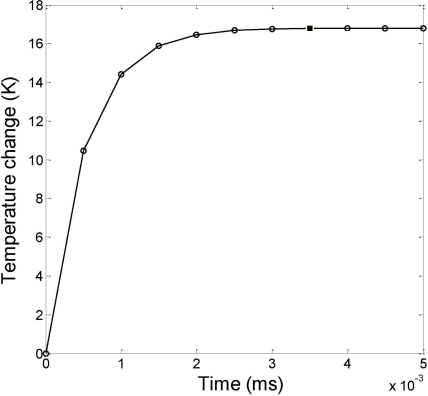
Transient response of the temperature on the cantilever beam simulated using Matlab.

Based on the material properties and dimensions, the temperature change due to the self-heating effect discussed above is found to be ∼17 °K using Equation (19) which is under a biasing voltage of 1.0 V that results in approximately 1 mA driving current. These results are further verified by the numerical simulations. The thermal transient response of the polysilicon resistor is plotted in Figure 6, as a result of Equation (19).

### 2.3. Device Simulation

**Figure 7.**
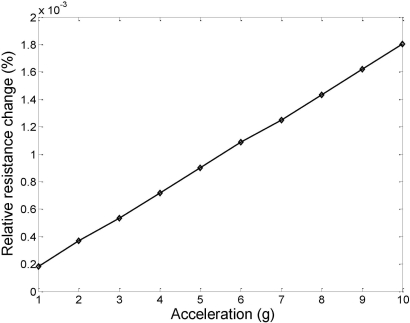
CoventorWare simulation of the piezoresistance change in longitudinal direction as a function of out-of-plane acceleration.

CoventorWare, a comprehensive finite element analysis (FEA) tool dedicated for MEMS design and simulation, is used to validate the relative resistance change of the piezo resistors design. From CoventorWare simulation, as shown in Figure 7, it is found that the relative piezoresistance change in longitudinal direction can be as high as 1.8 × 10^−4^%/g or 1.7 mΩ/g. The FEA simulation results are in good agreement with the theoretical calculation as shown in Figure 4.

**Figure 8.**
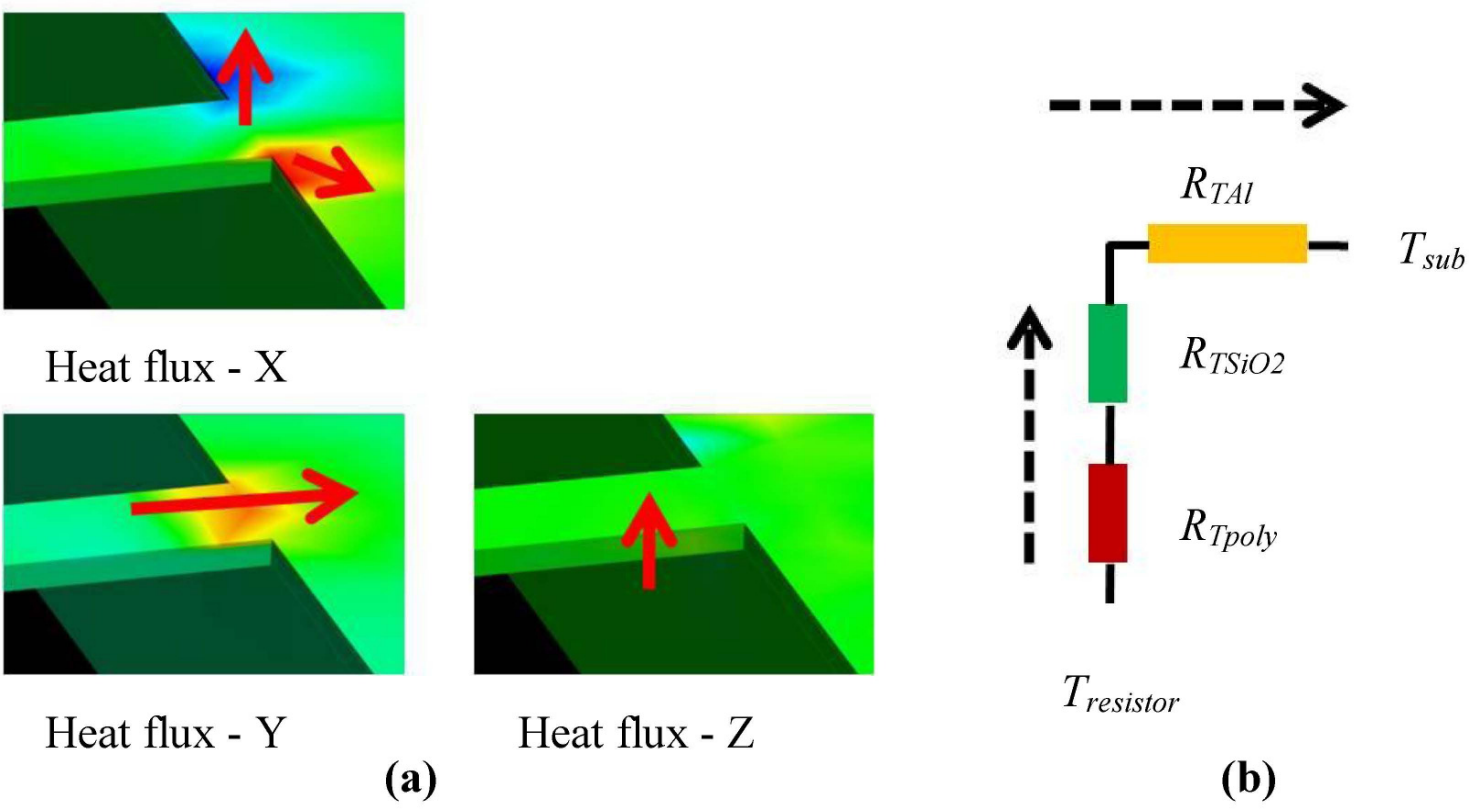
**(a)** CoventorWare simulation results showing the heat flux between the polysilicon sensors and the substrate; and **(b)** the equivalent thermal resistance in the system.

Figure 8 shows the CoventorWare simulation results of the heat flux distribution from the polysilicon sensing element to the substrate.

A 1 mA driving current is applied through the polysilicon resistor. From the contour representing the direction and value of the flux, a heat dissipation path of Z → Y → X can be derived, as predicted in Equation (20). The simulated self-heating caused temperature rise ∼12 °K. This result is in good agreement with the theoretical calculation. The relative change of resistance due to the self heating effect is 0.1639 %. This change is even larger than the resistance change due to acceleration as shown in Figure 7. It should be noted that the self heating is a universal effect, happening to all the resistors monotonously. Consequently this effect can be largely canceled due to the Wheatstone configuration of the polysilicon sensors. Yet it is noteworthy to gain insights into the sensing mechanism in this composite structured CMOS-MEMS sensor.

## 3. Device Fabrication

**Figure 9.**
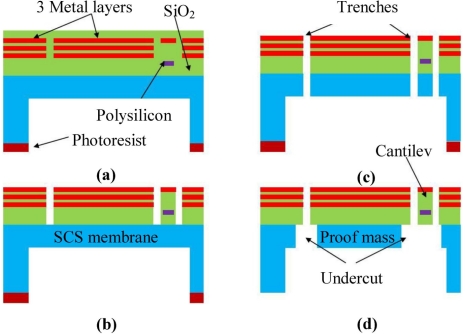
Post-CMOS microfabrication process flow.

The ON Semiconductor 0.5 μm CMOS technology has been utilized for CMOS fabrication of the sensor through MOSIS. The post-CMOS process, with a flow as illustrated in Figure 9, has been used to release the structure. The process starts with a back side selective DRIE process that produce a SCS membrane with a thickness of ∼40 μm, as illustrated in Figure 9(a). Anisotropic SiO_2_ RIE is then performed on front side of the device to open patterns of the composite beams and proof mass, as shown in Figure 9(b). Next, silicon DRIE process is used to etch through the structure, as in Figure 9(c). Finally, an isotropic silicon etching undercuts the silicon underneath the composites and releases the device, as illustrated in Figure 9(d). During the isotropic undercut process, a small portion of the proof mass and the substrate will also be undercut. This problem will not have a large effect to the sensor performance due to the large proof mass dimension. The backside photoresist used in the process can be removed by oxygen ashing.

**Figure 10.**
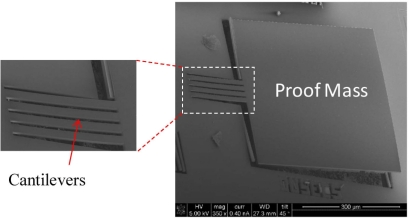
SEM image of the fabricated CMOS-MEMS accelerometer with the inset showing the composite beams where piezoresistors are located.

Figure 10 shows a SEM photograph of the fabricated sensor with inset showing close-up of the cantilever beams. The structure curling observed in Figure 10 is due to the residual stress existing among the CMOS thin films. This curling is much smaller than that in the thin film sensors [11].

## 4. Device Characterization

**Figure 11.**
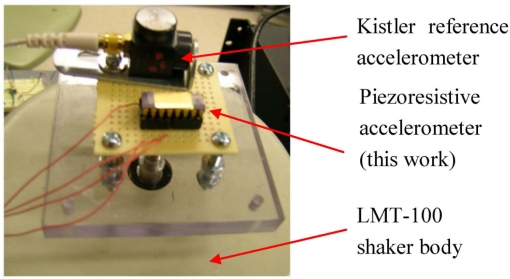
Test board on which the DUT and the reference accelerometer are mounted.

The resistance of the polysilicon resistor in each beam is measured as ∼1.32 kΩ. With a 1 mA biasing current, the sensor demonstrates a sensitivity of ∼0.077 mV/g prior to amplification, much larger than what were reported in [2,10,11]. The greatly increased sensitivity is mainly due to the large proof mass attached to the ends of composite cantilever beams. This attribute is particularly enabled by the bulk CMOS-MEMS microfabrication process we have developed, as described in reference [12]. It’s also noteworthy that due to the complete protection of polysilicon sensing elements in post-CMOS process, the device structure allows considerable process variations in microfabrication. The piezoresistors are immune to the slight over-etching in device release that is necessary for complete undercut of the beams. Other sensor characterizations are performed with an external instrumentation amplifier that has a voltage gain of 52 dB. In the dynamic test, a LMT-100 shaker from Ling Electronics is used to provide standard acceleration. A Kistler type 8692B50 accelerometer is used as a reference. It is calibrated using a 394C06 hand-held shaker from PCB Piezotronics, which generates a standard 1 g acceleration at a nominal frequency of 159.2 Hz. Figure 11 shows the test board on which the device under test (DUT) is assembled with the reference accelerometer. The DUT is packaged in a standard ceramic 16-pin DIP package. The board is screwed to the threaded pole of the shaker.

**Figure 12.**
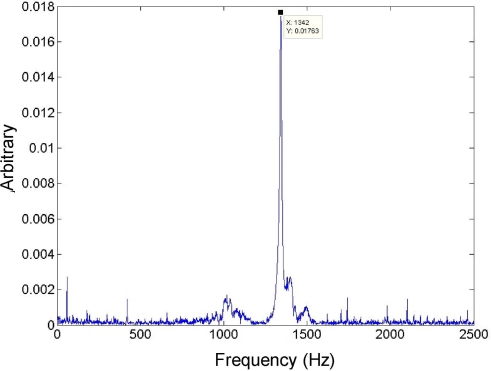
Spectrum of the sensor response to an impulse excitation.

The resonant frequency of the sensing element has been identified as 1.34 kHz through an impulse test. The dynamic response spectrum of the sensor is shown in Figure 12. The lower resonant frequency of the structure compared to the simulation result is caused by the following combined effects: the thicker proof mass than the designed value; the undercut around the composite beams; and the thickness reduction of the top aluminum layer resulted from the physical milling effect in the DRIE etching.

**Figure 13.**
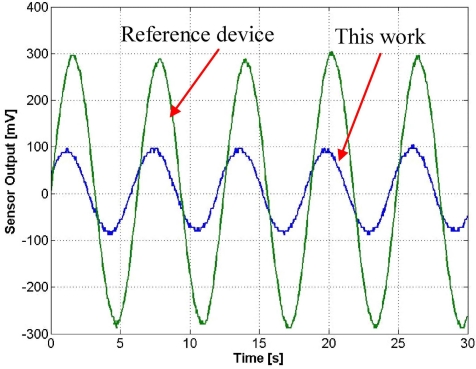
Waveforms of the DUT and reference accelerometer to a sinusoidal 3 g excitation.

**Figure 14.**
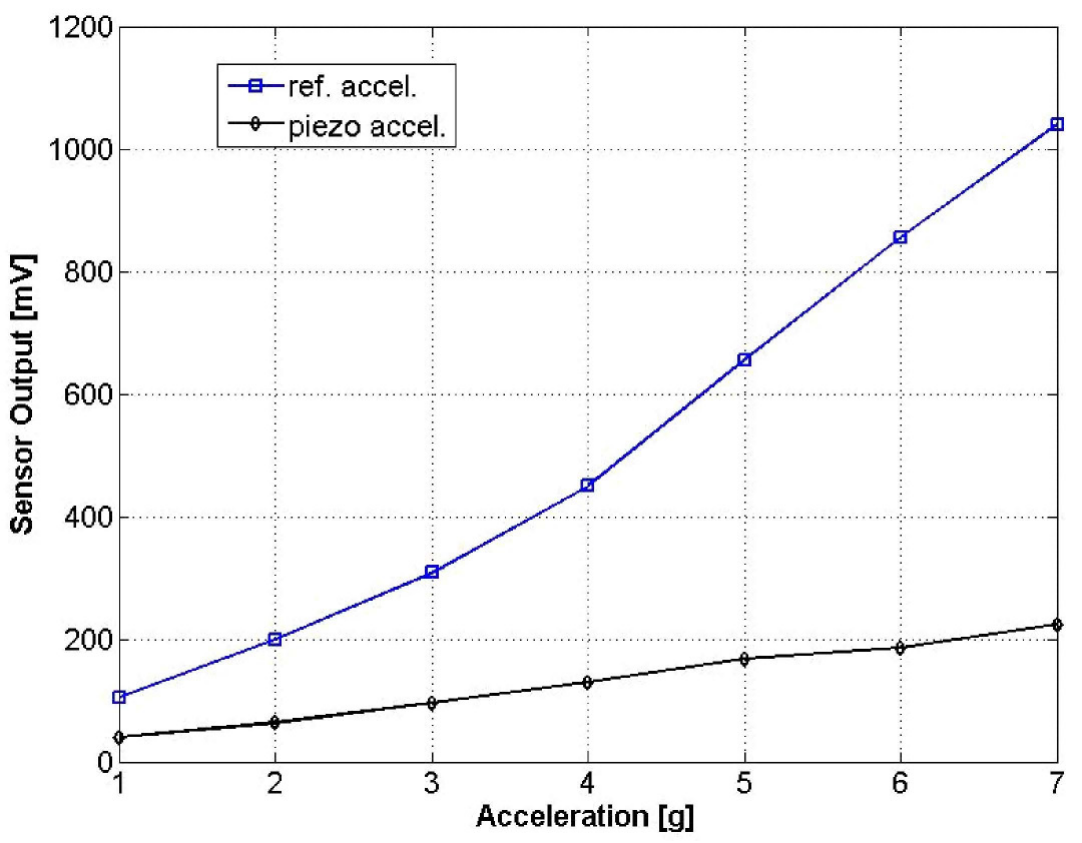
Sensor responses to accelerations ranging from 1 g to 7 g.

Figure 13 shows an output waveform under a 3 g excitation at 160 Hz. With a 52 dB amplification gain, the sensor produces an output of 192 mV_p − p_. The testing frequency of 160 Hz is chosen to be in consistence with the frequency at which the reference accelerometer has been calibrated. Figure 14 compares the output of the fabricated sensor with the reference accelerometer output in an acceleration range from 1 g to 7 g. The piezoresistive accelerometer reported in this work demonstrates a better linearity than the reference device.

The merits of the piezoresistive accelerometer demonstrated in this work are further summarized in Table 4. A comparison between the device in this work and that is reported in reference [10] is also made. It should be noted that in the device in reference [10], the CMOS thin film structures are fabricated using wet bulk micromachining process, which requires careful protection of the CMOS region in wet etching. Whereas the dry etching based process in this work has better process tolerance due to the complete protection of the polysilicon sensing element.

## 5. Conclusions

A low-cost, high-sensitivity z-axis CMOS-MEMS piezoresistive accelerometer with large proof mass has been successfully demonstrated. The accelerometer has a 0.077 mV/g mechanical sensitivity with a very low power consumption of ∼1.5 mW for operation. Common issues associated with CMOS-MEMS thin film accelerometers such as large structural curling and low sensitivity have been solved by incorporating SCS in the proof mass. The four cantilever beams employed in the sensor has significantly improved the sensor stability by allowing sorely the out-of-plane motion of the proof mass and minimizing the in-plane motion. Multiple CMOS metal layer permits flexible wiring of the sensing elements for full Wheatstone bridge configuration, which further increases the sensitivity of the accelerometer. While monolithic integration of amplification and signal conditioning circuits in progress, the demonstrated z-axis accelerometer provides a low cost solution for out-of-plane sensing that is normally more challenging for capacitive sensors. This device can find many applications such as in portable electronics.

## Figures and Tables

**Table 1.** Typical CMOS Layers Thickness in the ON 0.5 μm Technology Used.

**Layers**	**Thickness (μm)**
Single Crystal Silicon (SCS)	∼250
Field Oxide under Poly, *H_ox_*	0.4
Field Oxide under Metal 1	0.375
Gate Oxide	0.0135
Polysilicon, *t_poly_*	0.35
Metal, *H_Al_*	0.77
Boron-phosphorus-silicate-glass (BPSG)	0.7

**Table 2.** Sensor Dimension and Material Properties.

**Symbol**	**Description**	**Value**
Mechanical:		
*E_poly_*	Polysilicon Young’s modulus	160 GPa
*E*	Effective Young’s modulus of the beam	70 GPa
*m*	Proof mass weight	105 μg
Geometric:		
*W_b_*	Cantilever beam width	13 μm
*H_b_*	Cantilever thickness from polysilicon layer	4.2 μm
*L_b_*	Cantilever beam length	200 μm
*L_pm_*	Proof mass length	500 μm
*W_pm_*	Proof mass width	500 μm
*L_poly_*	Length of poly resistor	49.4 μm
*W_poly_*	Width of poly resistor	1.2 μm
Electrical:		
*ρ_s_*	Polysilicon sheet resistance	26.1 Ω/square
*α_poly_*	Polysilicon temperature coefficient of resistance (TCR)	2.1 × 10^−3^ K^−1^*
*ρ_poly_*	Polysilicon resistivity	9.14 Ωμm
Thermal:		
*κ_Al_*	Aluminum thermal conductivity	237 W/(K.m)
*κ_ox_*	SiO_2_ thermal conductivity	1.1 W/(K.m)
*κ_Si_*	Silicon thermal conductivity	170 W/(K.m)
*κ_poly_*	Polysilicon thermal conductivity	29 W/(K.m)

* Note: This data is obtained from the temperature characterization on the fabricated device.

**Table 3.** Calculated Thermal Resistances and Capacitances.

**Symbol**	**Description**	**Values**
*A_poly_*	Area of the Polysilicon resistor	5.93 × 10^−11^ m^2^
*A_ox_*	Area of the Polysilicon resistor	1.37 × 10^−10^ m^2^
*W_Al_*	Area of the oxide	13.4 × 10^−6^ m
*L_Al_*	Width of the metal	10.2 × 10^−6^ m
*R_Tpoly_*	Thermal resistance of polysilicon layer	233 K/W
*R_TSiO2_*	Thermal resistance of silicon dioxide layer	1.2 × 104 K/W
*R_TAl_*	Thermal resistance of aluminum layer	4.6 × 103 K/W
*C_Tpoly_*	Thermal capacitance of polysilicon layer	3.88 × 10^−11^ J/K
*C_TSiO2_*	Thermal capacitance of silicon dioxide layer	3.80 × 10^−10^ J/K
*C_TAl_*	Thermal capacitance of aluminum layer	2.32 × 10^−10^ J/K
*R_T_*	Total thermal resistance	1.7 × 10^4^ K/W
*C_T_*	Total thermal capacitance	3.1 × 10^−11^ J/K

**Table 4.** Summary of the merits of the demonstrated device and comparisons with the device demonstrated in Reference [10].

**Specifications**	**This work**	**Device in Reference [10]**
Proof mass	230 μg	0.96 μg
Cantilever beam length	200 μm	500 μm
Proof mass size	500 μm × 500 μm	280 μm × 280 μm
Sensitivity	∼77 μV/g	∼2.0 μV/g
Power consumption	1.5 mW	N/A
Fabrication process	DRIE and RIE	Front side wet bulk micromachining
Process tolerance	Good	Need front side protection
Structure curling	Mediocre	Large
